# Genital warts and cervical neoplasia: an epidemiological study.

**DOI:** 10.1038/bjc.1983.243

**Published:** 1983-11

**Authors:** S. Franceschi, R. Doll, J. Gallwey, C. La Vecchia, R. Peto, A. I. Spriggs

## Abstract

Cervical carcinoma and cervical intra-epithelial neoplasia (CIN) are likely to be associated with all sexually transmitted diseases (STDs). To help discover which (if any) of the recognised STDs might actually cause these conditions, a key question is whether one particular such association is much stronger than the others. The present study is therefore only of women newly attending an STD clinic, and compares the prevalences of cytological abnormalities of the cervix among 415 women attending with genital warts, 135 with genital herpes, and 458 with trichomoniasis or gonorrhoea. Significantly more genital wart patients (8.1%) than trichomoniasis or gonorrhoea patients (1.9%) showed dyskaryotic changes (adjusted relative risk (RR) = 5.8 with 95% limits 2.5-13.5) at, or a few months before, first attendance, while no excess whatever was seen in women with genital herpes. Moreover, half the women had a subsequent smear (at an average of 3-4 years after first attendance) and, although the diagnosis at first attendance was not related to the onset rate of dyskaryotic changes observed in these subsequent smears, it was related to the onset rate of grade III cervical intra-epithelial neoplasia (CIN III), which was found in 7 previous genital wart patients, in 2 previous trichomonas patients, but in 0 previous genital herpes patients. Thus, our findings suggest that herpes is not directly relevant to dyskaryotic change, but that one or more of the human papilloma viruses that cause genital warts may be.


*Br. J. Cancer* (1983), **48**, 621–628

### Genital warts and cervical neoplasia: An epidemiological study

S. Franceschi[1*], R. Doll[1], J. Gallwey[2], C. La Vecchia[1*], R. Peto[3] & A. I. Spriggs[4]

[1]*ICRF Cancer Epidemiology & Clinical Trials Unit, Radcliffe Infirmary,* [2]*Harrison Department, Radcliffe Infirmary,* [3]*ICRF Cancer Studies Unit, Nuffield Department of Clinical Medicine, Radcliffe Infirmary, Oxford OX2 6HE,* [4]*Laboratory of Clinical Cytology, Churchill Hospital, Oxford.*

**Summary** Cervical carcinoma and cervical intra-epithelial neoplasia (CIN) are likely to be associated with all sexually transmitted diseases (STDs). To help discover which (if any) of the recognised STDs might actually cause these conditions, a key question is whether one particular such association is much stronger than the others. The present study is therefore only of women newly attending an STD clinic, and compares the prevalences of cytological abnormalities of the cervix among 415 women attending with genital warts, 135 with genital herpes, and 458 with trichomoniasis or gonorrhoea. Significantly more genital wart patients (8.1%) than trichomoniasis or gonorrhoea patients (1.9%) showed dyskaryotic changes (adjusted relative risk (RR) = 5.8 with 95% limits 2.5–13.5) at, or a few months before, first attendance, while no excess whatever was seen in women with genital herpes. Moreover, half the women had a subsequent smear (at an average of 3–4 years after first attendance) and, although the diagnosis at first attendance was not related to the onset rate of dyskaryotic changes observed in these subsequent smears, it was related to the onset rate of grade III cervical intra-epithelial neoplasia (CIN III), which was found in 7 previous genital wart patients, in 2 previous trichomonas patients, but in 0 previous genital herpes patients. Thus, our findings suggest that herpes is not directly relevant to dyskaryotic change, but that one or more of the human papilloma viruses that cause genital warts may be.

Although it has long been suspected that cervical cancer is of venereal origin (Kessler, 1976), it has proved remarkably difficult to determine whether any of the known sexually transmitted infective agents are responsible for it, and, if so, which one chiefly is. This is partly because the patterns of behaviour, either by the woman or by her partner(s), that predispose to any one particular type of sexually transmitted disease (STD) are likely also to predispose to others, producing strong but non-causal associations of cervical cancer risk with many different STDs. Any truly causative STD must, therefore, show not merely an association with cervical cancer, but in particular an association that is much stronger than that for other STDs.

The tendency for all STDs to be associated with each other, and hence with cervical cancer, has not always been properly allowed for, and it alone probably accounts for the reported associations of the neoplasm with syphilis, gonorrhoea, and trichomoniasis (Alexander, 1973). If, however, the causative agent is not some hitherto unrecognised organism, then the most plausible candidates at present are perhaps herpes simplex virus type II (HSV2) or some type(s) of human papilloma virus (HPV).

At first sight, HSV2 fits the requirements for venereal carcinogen rather well. Women with cervical cancer have consistently been shown to have higher levels of antibodies to various HSV2 antigens than controls (Rawls & Adam, 1977; Aurelian, 1980) and HSV2-specific RNA, though not DNA (Eglin *et al*, 1981), has occasionally been detected in the nuclei of cells from cervical carcinomas. Nevertheless, some inconsistencies still remain, and in a recent review article (Waterson, 1982) the conclusion about HSV2 was merely that "much epidemiological work has so far failed to transform suspicion into certainty".

The other plausible candidate, HPV, should really be thought of as a set of candidate viruses, for it includes several distinct viral types (Tooze, 1981), all of which are small DNA viruses. In humans, the type(s) chiefly responsible for warts on the hands differ from the venereally transmitted type(s) chiefly responsible for genital warts (GW) (*condylomata acuminata*); but it is not yet known how many distinct types and subtypes of venereally transmitted HPV exist. On the cervix itself HPV infection can induce lesions ranging from recently recognised subclinical changes—involving, on cytological examination of a cervical smear, features such as "koilocytotic atypia" (nuclear enlargment and irregularity, with a perinuclear

*Present address: Mario Negri Institute for Pharmacological Research, Milano, Italy.
Correspondence: R. Peto.
Received 18 June 1983; accepted 22 July 1983.

© The Macmillan Press Ltd., 1983.

halo) and/or, on colposcopic examination of the cervix itself, asymptomatic features such as "flat" warts—to the classical findings of raised (i.e. papillomatous) warts (Meisels *et al.*, 1977). However, although papillomatous warts provide specific evidence of HPV infection, it is not known to what extent koilocytotic atypia does likewise.

Malignant transformation has been reported in dozens of papillomatous GW in various parts of the male and female genital tract (Boxer & Skinner, 1977) and also in flat warts (Shokri-Tabibzadeh, 1981), and at least some of the few genital neoplasms so far investigated (2 vulvar carcinomas, 6 cervical carcinomas and 3 cervical carcinomas *in situ*) contained papilloma virus DNA (zur Hausen, 1982).*

### *Difficulties in assessing HPV infection of the cervix*

When attention was restricted to clinical papillomatous warts, HPV infection of the cervix itself was thought to be very rare. If, however, all of its possible manifestations (including flat warts and/or koilocytotic atypia) are included, then among large series of asymptomatic women attending cervical screening clinics at least 1% appear to have cytological evidence suggestive of such infections (Reid *et al.*, 1980; Meisels *et al.*, 1977). Moreover among women with frank warts elsewhere in the genital tract, such cytological or histological changes in the cervix may be detected in 10–50% (Jagella & Stegner, 1974; Purola & Savia, 1977; Baggish, 1980). All of these percentages may eventually require substantial revision, however, for neither the sensitivity nor the specificity of cytological findings have yet been established as indicators of infection of the cervix by one or other type of HPV.

Indeed, the difficulty of determining reliably exactly who has (or has had) genital HPV (leave alone of any particular type) is a serious obstacle to progress in this field. Even in infected cells, viral particles—detectable by electron microscopy or by immunoperoxidase techniques—may be uncommon and although serum antibodies to genital HPV infection do form, they cannot at present be distinguished reliably from antibodies to the widely prevalent common warts of other parts of the body, such as the hands or feet. DNA probes for certain HPV types have recently emerged, but it remains to be seen how convenient and reliable tests based on these will be.

*In addition, Durst, Gissman, Ickenberg, and Zur Hausen (personal communication) have recently discovered what appears to be a new type of HPV (tentatively designated HPV16), evidence for which has been found in about half of the first few dozen cervical carcinomas examined.

Using histological methods to classify the cervical epithelium, Reid *et al.* (1982) reported that out of 80 women with cervical neoplastic changes (including 40 with evidence of invasion) 73 showed "subclinical papillomavirus infection" (SPI) while the remaining 7 showed "suspicious SPI". By contrast, 60/80 control cases were negative, 10 showed "suspicious SPI" and only 10 satisfied the full criteria for "SPI". If Reid's findings, or something like them, could be reproduced using more direct measurements of (preferably type-specific) HPV infection then they would provide strong evidence that HPV is indeed an important cause of cervical cancer. However, other workers, using different criteria, have not reported such extreme associations (Syrjanen *et al.*, 1981), and it is still possible that the changes referred to as "SPI" are due in part to some different viral infection, or to some indirect effects of the neoplasms themselves or of the preneoplastic conditions from which they arose. In short, we have no grounds for knowing the extent to which the cytological features of "SPI" are specific for HPV infection. Therefore, until reliable methods are available for the detection of HPV in cervical cells, a reliable diagnosis of infection can (in the absence of immunochemical or EM investigations) be made only in the presence of warts. At the ages when these are common, however, neoplastic changes in the cervix (carcinoma-*in-situ* or worse) are rare and substantial amounts of information can be obtained only for the lesser degrees of dysplasia, as evidenced in smears by "dyskaryosis". In the present study we have, therefore, sought to establish whether dyskaryosis is associated any more strongly with clinical GW than with genital herpes or other venereal infections.

### Patients and methods

### *Patients*

From the outpatients list of the Sexually Transmitted Diseases Clinic at the Radcliffe Infirmary, Oxford, women were selected who: (i) first attended in 1972–8; (ii) were white and resident in Oxfordshire; (iii) had not previously undergone hysterectomy; and (iv) had a diagnosis on first attendance of one of (a) genital warts (GW), (b) genital herpes simplex virus infection (HSV), or (c) Trichomonas vaginalis (TV) or gonorrhoea (GR). All women at first attendance for GW (415) and HSV (135) were included while a 50% sample was chosen (by alternation) of patients with TV (216) and GR (242). The HSV group included 19 patients with HSV1 infection, 90 with HSV2, and 26 with HSV of unspecified type.

Alternate women attending for TV or GR were chosen for investigation, rather than all the women attending for one or other disease, in the hope of obtaining a more representative sample of women at risk of developing STDs than might be obtained if the comparison group were limited to one disease only. In the event, the results obtained for women in the two categories were closely similar and they have, therefore, been treated as constituting a single group for comparison in all the analyses.

Information was also sought on age, date of birth, occupation, reproductive history, contraceptive practice, sexual behaviour over the 6 preceding months, and subsequent attendance for recurrences of venereal diseases. Table I shows that, compared to the other women, GW patients were quite different. They were younger, more were students, more were employed in clerical jobs, more were nulliparous, and more were users of oral contraceptives. In addition, they had on the average had fewer partners during the previous six months. Women with herpetic lesions showed somewhat intermediate features, but were rather similar to GW patients in their occupations and numbers of partners.

Many women (49.9% of GW, 51.9% of HSV

**Table I** Characteristics of 1,008 women attending Oxford STD Clinic in relation to diagnosis of venereal disease.

| | *Genital Warts* (*415 women*) | | *Genital Herpes* (*135 women*) | | *Trichomonas or Gonorrhoea* (*458 women*) |
|---|---|---|---|---|---|
| | (%) | *Chi-square*[1] *test statistic* | (%) | *Chi-square*[1] *test statistic* | (%) |
| *Age* (years) | | | | | |
| ≤19 | 43.6 | 24.44 | 30.4 | | 32.3 |
| 20–24 | 37.3 | (df=1; | 34.8 | (NS) | 33.2 |
| ≥25 | 19.0 | $P<0.001$) | 34.8 | | 34.5 |
| *Occupation* | | | | | |
| Student | 29.6 | | 25.9 | | 12.7 |
| Clerical worker | 32.0 | | 30.4 | | 26.6 |
| Manual worker or sales assistant | 22.2 | 60.48 | 21.5 | 17.80 | 27.3 |
| Housewife or | | (df=3; | | (df=3; | |
| unemployed | 16.1 | $P<0.001$) | 22.2 | $P<0.001$) | 33.4 |
| *Marital status* | | | | | |
| Single | 76.1 | (NS) | 65.3 | (NS) | 62.7 |
| Ever-married | 23.9 | | 34.8 | | 37.3 |
| *No. of children* | | | | | |
| None | 86.0 | 16.13[2] | 72.6 | (NS) | 64.3[3] |
| ≥1 | 14.0 | | 27.4 | | 35.7 |
| | | ($P<0.001$) | | | |
| *Oral contraceptives*[4] | | | | | |
| User | 66.3 | 5.45 | 60.0 | (NS) | 55.2 |
| Non-user | 33.7 | | 40.0 | | 44.8 |
| | | ($P<0.02$) | | | |
| *Recent partners*[4] | | | | | |
| 1 | 65.8 | 22.70[2] | 63.7 | 4.75[2] | 55.9 |
| 2 | 27.7 | (df=1; | 26.7 | (df=1; | 25.8 |
| ≥3 | 6.5 | $P<0.001$) | 9.6 | $P<0.03$) | 18.3 |

[1]$\chi^2$ test for heterogeneity or, where appropriate, trend, comparing percentage distribution of characteristic with that among women with trichomonas or gonorrhoea. NS = not significant.

[2]Adjusted for age (≤19; 20–24; ≥25 years) and occupation (student/clerical vs. others) by the Mantel Haenszel procedure.

[3]Excluding one woman with unrecorded parity.

[4]In the 6 months preceding attendance.

and 38.2% of TV/GR) were also suffering from other genital infections at first attendance, chiefly candidiasis or unspecific vaginal discharge, and many (23.4% of GW, 30.1% of HSV and 30.3% of TV/GR) were referred back to the STD clinic during the subsequent 1–10 years, either for the same disease or for a different condition. 9.9% of the GW patients were referred back for a recurrence and 5.9% of HSV and 3.7% of TV/GR women were referred back for a first onset of GW.

Among 1,008 women included in the present study, 92.3% of GW, 90.3% of HSV and 89.7% of TV/GR women had a cervical smear taken at first attendance or had had one within the previous 6 months, and the findings of these smears are the chief subject of the present study. In addition, the records of the Cytology Department of the Churchill Hospital, Oxford, were scanned for previous and subsequent smear results on these women, whether from the STD clinic or elsewhere (general practitioners, family planning clinics, etc.). These records contained reports on all cervical smears examined cytologically in the area and provided our only means of follow up, because the policy of the STD Department did not permit patients to be recalled for the purpose of the study. In 52.7% of GW, 52.5% of HSV and 51.6% of TV/GR patients, subsequent smear(s) were identified, and where this happened the mean numbers were respectively 2.0, 2.0 and 1.9 subsequent smears at 12–120 (means respectively 43, 38 and 47) months after entry. In addition, some information on the cytological status of the cervix more than 6 months before referral to the STD clinic was found in 16.9% of GW, 24.4% of HSV and 25.3% of other women.

### Cytology

All smears were reviewed by one of us (AIS) and classified into 4 groups: (i) normal, (ii) inflammatory, (iii) superficial (mild) dyskaryosis (large, hyperchromatic, irregular nuclei in cells with normal or near-normal maturation, corresponding to mild/moderate dysplasia or CIN I–II), and (iv) parabasal dyskaryosis (poor cytoplasmic differentiation, corresponding to severe dysplasia/carcinoma *in situ* or CIN III) (Spriggs *et al.*, 1978). Separation of "koilocytotic atypia" from dyskaryosis was not considered reliable but characteristics pointing to HPV infection (koilocytosis, binucleation and multinucleation, and presence of orange-stained cells with pyknotic nuclei) were assessed "blindly" in all dyskaryotic smears. Biopsy, electrocoagulation diathermy or other treatment of the cervix was not considered for mild dyskaryosis unless the lesion persisted for at least 6–12 months. Since in most cases of dyskaryosis there was spontaneous "regression" to a negative smear, no biopsy was done and so no histological diagnosis is reported.

### Statistics

Test of the statistical significance of any apparent differences were based on standard chi-square tests for trend or for heterogeneity (with correction for continuity in the particular "2 × 2" case of a 2-group comparison of a 2-level quantity). Differences between cases and controls in age and occupation were adjusted for by the method of Mantel (1963) or Mantel & Haenszel (1959). Odds ratios (as estimates of relative risks, RR) and their approximate 95% confidence intervals (CI) were also calculated (Miettinen, 1976).

### Results

### Cytological findings at first attendance

Significantly more women with GW than women with other STDs presented with some degree of dyskaryosis (Table II). Among screened patients, 31 (8.1%) of the GW series were found to have superficial dyskaryosis compared to 8 (1.9%) of the women with TV/GR. The estimated relative risk (RR) for the occurrence of dyskaryotic smears in GW compared to TV/GR was 4.4 (95% CI: 2.1–14.7). After adjustment for age and occupational group, this relative risk increased to 5.8 (95% CI: 2.5–13.5). Clinical HPV infection of the cervix was associated with an even higher rate of superficial dyskaryosis (8/36 vs 23/347, $\chi^2_1 = 8.65$, $P < 0.01$) and for this group of patients the estimated relative risk would have been greater still. By contrast, among screened patients with herpetic lesions, only 2 (1.6%) presented with dyskaryosis (RR = 0.8).

In line with the general policy of the clinic, treatment (including cervical biopsy) was deferred for 6–12 months in all 40 women showing mild dyskaryosis. The only woman with severe dyskaryosis (a TV patient) was biopsied immediately and this revealed a microinvasive cervical carcinoma. She was treated by hysterectomy.

### Previous cytological findings

Some degree of dyskaryosis has been reported before clinical evidence of venereal disease in 6 (8.6%) of 70 GW patients (6 years before in one case and 2 years before in the others), in none of the 33 HSV patients, and in 4 (3.4%) of the 116 TV/GR patients (1, 2 and 3 years before the first attendance) (Table III). Although this excess is similar in direction to that observed at entry to the

**Table II** Cytological findings at, or within 6 months before, first attendance at Oxford STD Clinic, in relation to diagnosis of venereal disease.

| | *Genital Warts* | | *Genital Herpes* | | *Other (Trichomonas/Gonorrhoea)* | |
|---|---|---|---|---|---|---|
| *Cytological finding* | *No. of women* | *% of smears* | *No. of women* | *% of smears* | *No. of women* | *% of smears* |
| No smear taken | 32 | — | 13 | — | 47 | — |
| Negative | 296 | (77.3) | 97 | (79.5) | 296 | (72.0) |
| Inflammatory | 56 | (14.6) | 23 | (19.9) | 107 | (26.0) |
| Superficial dyskaryosis or worse[1] | 31 | (8.1) | 2 | (1.6) | 8 | (1.9) |
| RR (and 95% CI) for dyskaryosis or worse | 4.4[2] (2.1–14.7) | | 0.8 (0.1–7.1) | | (1.0)[3] | |

[1]The only "worse" than superficial dyskaryosis finding was a woman aged 44 with TV and microinvasive cervical carcinoma treated by hysterectomy.
[2]RR (adjusted for age and occupational group by the Mantel-Haenszel procedure) = 5.8 (95% CI: 2.5–13.5).
[3]Reference category.

**Table III** Cytological findings more than 6 months before first attendance at Oxford STD Clinic, in relation to eventual diagnosis of venereal disease at the Clinic.

| | *Genital Warts* | | *Genital Herpes* | | *Other (Trichomonas/Gonorrhoea)* | |
|---|---|---|---|---|---|---|
| *Cytological finding (more than 6 months earlier)* | *No. of women* | *% of smears* | *No. of women* | *% of smears* | *No. of women* | *% of smears* |
| No smear available | 345 | — | 102 | — | 342 | — |
| Negative | 64 | (91.4) | 33 | (100.0) | 112 | (96.6) |
| Superficial dyskaryosis or worse | 6 | (8.6) | 0 | — | 4 | (3.4) |
| RR (and 95% CI) for dyskaryosis or worse | 2.6[2] (0.5–13.4) | | | | (1.0)[3] | |

[1]On the average, 2 years earlier.
[2]RR (adjusted for age by the Mantel Haenszel procedure) = 2.5. (95% CI: 0.5–13.1).
[3]Reference category.

study, it is based on such small numbers that it is not statistically significant (and would not be so even if the HSV patients were pooled with the TV and GR patients).

Among the 6 GW women and the 4 TV/GR women who had had dyskaryosis in the years preceding the diagnosis of the specified venereal disease, 3 and one respectively still showed it at entrance.

*Subsequent cytological findings*

The percentages of women who were re-examined, the mean numbers of smears, and the mean lengths of follow-up were very similar in the 3 groups (see **Patients** above). Surprisingly, in view of the findings at first attendance, the proportions with no cytological abnormality at entry who developed abnormalities were also rather similar in each group

(9.8% in 174 GW, 7.9% in 63 HSV, and 9.0% in 201 TV/GR women). Among women previously having superficial dyskaryosis, the condition persisted or worsened in 12/28 GW and 4/6 TV/GR women (including in the 4 the women who initially showed micro-invasive cervical carcinoma and had a hysterectomy) and regressed to normal in the one such women who had presented with HSV.

Some women, with or without clinical GW initially, presented with GW 1–10 years later, and all the available data on the occurrence of genital warts and dyskaryosis at first attendance or later are summarised in Table IV. These include observations on 16 women who had their first smear taken only a year or more after their first attendance. Dyskaryosis was observed in one woman in this last group who presented initially with GW and returned to the clinic 7 years later for another reason.

*Histological findings*

Histological examination of cervical biopsies or larger specimens at cone biopsy or hysterectomy, which was carried out only in cases of "parabasal cell dyskaryosis" or if "superficial cell dyskaryosis" persisted for 6–12 months, revealed 2 cases of micro-invasive cancer and 7 cases of grade III cervical intra-epithelial carcinoma. Six of the latter occurred in women who attended with GW (4 in women aged 21, 22, 22, and 26 years who returned to the clinic more than a year after having had negative smears at their first attendance and 2 in women aged 22 and 23 years who returned later having originally had mildly dyskaryotic smears) and only one occurred in any other group (in a woman aged 28 years who presented with TV, was found to have mild dyskaryosis, and subsequently returned with a persisting lesion). The cases of micro-invasive cancer occurred in (i) a woman aged 26 years who failed to have a smear taken on her initial visit to the clinic for GW 7 years previously and had returned for another reason, and (ii) the women aged 44 years at her first visit for TV, who has been referred to previously. A micro-invasive carcinoma of the vulva was also found in one of the women with GW who were found to have CIN III of the cervix at a subsequent attendance. The detailed distribution of all these cases is shown in the footnotes to Table IV.

*Changes in cytological smears "suggestive" of HPV infection*

Among women with dyskaryotic changes, cytological features that have been regarded as suggestive of HPV infection (see above) were present in 17/31 GW, 1/2 HSV and 2/8 other women at entry and in 7/28 GW, 2/5 HSV, and 5/20 other women at follow up. In our series, these features did not seem to be specific for patients with HPV infections unless a high proportion of

**Table IV** Cytotological findings at, and subsequently to first attendance at Oxford STDs Clinic, in relation to initial disease and subsequent development of genital warts ($N=1008$).

| | *Genital Warts at entry* | | | *Other at entry (Herpes/Trichomonas/Gonorrhoea)* | | |
|---|---|---|---|---|---|---|
| | *Negative smear at entry* | *Dyskaryotic smear at entry* | *No smear at entry* | *Negative smear at entry* | *Dyskaryotic smear at entry* | *No smear at entry* |
| *Subsequent smear(s)* | | | | | | |
| (a) *Presented with genital warts* | | | | | | |
| (1) Negative smear | 21 | 2 | 0 | 13 | 0 | 1 |
| (2) Dyskaryotic smear | 3 (1)[2] | 3 | 0 | 2 | 0 | 0 |
| (b) *Other reason*[1] | | | | | | |
| (i) Negative smear | 136 | 14 | 3 | 228 | 3 | 11 |
| (ii) Dyskaryotic smear | 14 (3)[2] | 9 (2)[2] | 1 (1)[2] | 21 | 4 (2)[2] | 0 |
| (c) *No smear since entry* | | | | | | |
| | 178 | 3 | 28 | 259 | 3 | 48 |
| Total | 352 | 31 | 32 | 523 | 10 | 60 |

[1]Includes attendance at STD Clinic for other venereal diseases and attendance at all other clinics.

[2]The figures in brackets give the numbers of the women in each cell of this table among whom CIN III or microinvasive cancer was found after first attendance at the STD clinic. They include the woman with TV who had a hysterectomy for microinvasive cancer at her first attendance.

women with other venereal infections also have subclinical wart virus infection.

### Discussion

There are two main sources of difficulty in interpreting these data. The first is purely epidemiological; because of the effects of the play of chance when only small numbers of events are studied, because of the crudeness of our measures of HPV infection, and because of the incompleteness of the available data (many women did not have a prior or a subsequent smear, and even those who did had only one or a few such smears, perhaps with long intervals between them), there is uncertainty about both the strength and the time course of the association between HPV infection and dyskaryosis. Indeed, the follow-up data, in which the onset rate of dyskaryosis is similar in GW, HSV, and TV/GR patients, stand in such marked contrast to the entry data, where the prevalence of dyskaryosis was far lower in the HSV and TV/GR patients than in the GW patients, that no plausible model springs to mind that can embrace both sets of observations without assuming that one, or other, or both have been substantially distorted by the play of chance. Perhaps the most plausible interpretation of the available data is that (i) there is no indication whatever of any association of herpes simplex infection with dyskaryosis or carcinoma-*in-situ,* and that (ii) there is strong evidence, chiefly from the entry data, for an association of HPV infection with dyskaryosis that is closer than the associations of certain other sexually transmitted diseases with dyskaryosis. But in the light of the follow-up data, the true association (i.e. the association that would be found at entry in a far larger study) is probably less close than is suggested by the present entry data. Further such studies could clarify this point. We must note, that women who attended an STD clinic for the selected conditions may not be typical of the entire population of affected women, nor may those who had had previous cervical smears or had them taken again during the follow-up period be typical of those who had smears taken on their first attendance at the clinic. It is unlikely, however, that any such atypicality could have materially affected the comparisons between the three STD groups with which we are concerned, as the percentages of those who had smears were so similar (see **Patients** above).

The second source of difficulty is biological, and is concerned with the nature of the dyskaryotic lesions that we have shown to be associated with genital warts. There are terminological difficulties (for example, many others might use the word dysplastic instead of dyskaryotic), but these are less important than the difficulties that derive from ignorance of the usual natural history of such lesions. Particularly, even if it were accepted that some type(s) of dysplastic change could predispose to malignant change, how homogeneous a category is "dysplastic lesions"? Unfortunately, no follow-up study dealing with different types of dysplastic lesion has been large and long enough to establish the existence of different types of dysplasia that differ in their natural history (Boon & Fox, 1981; Meisels *et al.,* 1981) and chromosomal analysis of mild dysplasias has not been possible because of their low mitotic activity (Spriggs, 1974).

If it is accepted that some types of HPV infection can cause "koilocytotic atypia" (Koss, 1979; Meisels *et al.,* 1977) then, since such lesions will often* be classified as "dysplastic", HPV can indeed cause at least one category of "dysplasia". But, is this category of dysplasia that is produced by HPV infection one that has no important relationship to malignant change? If so, HPV may be of little importance. Conversely, if dysplastic (or, more strictly, dyskaryotic) changes constitute a biological continuum, and if they can predispose to malignant change, then our evidence that HPV is a major cause of dysplasia tends to incriminate one or more of the types of HPV as being a likely cause (though not necessarily the only important cause) of cervical cancer. An indication that this may indeed be the case is provided by the observation (Table IV) that 7/206 women with an initial diagnosis of genital warts progressed to a grade III cervical intra-epithelial neoplasm (CIN III), as opposed to only 1/282 other women (excluding the women who initially underwent hysterectomy). These numbers are small, however, and the follow-up is incomplete; moreover, although CIN III appears to be a considerably more sinister lesion than those characterised only by superficial dyskaryosis, it is still not a malignant neoplasm, so some reservations about its relevance may also be justified.

Although these uncertainties are substantial, they do not entirely eclipse our findings. The epidemiological evidence that cervical cancer is generally caused by a venereally transmitted infective agent is overwhelming, and laboratory and

---

*In our series, it should be noted the presence of "Koilocytotic atypia" was not regarded as sufficient evidence to apply the term dyskaryosis (and, when dyskaryosis was reported, the proportion of cases in which koilocytotic atypia was apparent was only very slightly higher among GW patients than among TV or HSV patients).

other evidence suggests that herpes simplex and/or some types of human papilloma virus might be chiefly responsible (Zur Hausen, 1982), but does not yet point strongly to either. Whatever other conclusions may be drawn from our study, it most certainly does not show any association between genital herpes and either the immediate onset of dyskaryosis, or the onset over the next few years of CIN III. Our data weigh, therefore, quite heavily against any role for the common type(s) of genital herpes in causing dyskaryotic, and hence perhaps malignant, changes in the uterine cervix. Consequently, our data indict HPV not only directly, by showing its association with dyskaryosis and suggesting its association with CIN III, but also indirectly, by producing evidence against the relevance of the most plausible alternative to HPV as a causative factor of cervical cancer. If, however, it proves that only some types of HPV are responsible, progress is likely to be slow until they can readily be detected from others.

We wish to thank all the doctors, nurses and administrative staff working at the Harrison department, Radcliffe Infirmary, and the Cytology department, Churchill Hospital, Oxford. During this study SF and CLV were in receipt of a Research Training Fellowship awarded by the Italian Labour Ministry and EEC.

### References

ALEXANDER, E.R. (1973). Possible etiologies of cancer of the cervix other than herpesvirus. *Cancer Res.*, **33**, 1485.

AURELIAN, L., JARIWALLA, R.J., DONNENBERG, A.D. & SHERIDAN, J.F. (1980). Herpes simplex virus type 2 and cervix cancer: Perspectives on the possibility of a causal relationship. In: *The Role of Viruses in Human Cancer* (Eds. Giraldo & Beth). Elsevier North-Holland Inc. p. 75.

BAGGISH, M.S. (1980). Carbon dioxide laser treatment for condylomata acuminata venereal infections. *Obst. Gynecol.*, **55**, 711.

BOON, M.E. & FOX, C.H. (1981). Simultaneous condyloma acuminatum and dysplasia of the uterine cervix. *Acta Cytol.* **25**, 393.

BOXER, R.J. & SKINNER, D.G. (1977). Condylomata acuminata and squamous cell carcinoma. *Urology*, **9**, 72.

EGLIN, R.P., SHARP, F. MACLEAN, A.B., MACNAB, J.C.M., CLEMENTS, J.B. & WILKIE, N.M. (1981). Detection of RNA complementary to herpes simplex virus DNA in human cervical squamous cell neoplasms. *Cancer Res.*, **41**, 3597.

JAGELLA, H.P. & STEGNER, H.E. (1974). Zur Dignitat der Condylomata Acuminata. *Arch. Gynakol.*, **216**, 119.

KESSLER, I.I. (1976). Human cervical cancer as a venereal disease. *Cancer Res.*, **36**, 783.

KOSS, L.A. (1979). *Diagnostic Cytology and its Histopathologic Basis* (3rd edition). Lippincott, Philadelphia.

MANTEL, N. (1963). Chi-square tests with one degree of freedom; extension of the Mantel-Haenszel procedure. *J. Am. Stat. Assoc.*, **58**, 690.

MANTEL, N. & HAENSZEL, W. (1959). Statistical aspects of the analysis of data from retrospective studies of disease. *J. Natl Cancer Inst.*, **22**, 719.

MEISELS, A., FORTIN, R. & ROY, M. (1977). Condylomatous lesions of the cervix. II. Cytologic, colposcopic and histopathologic study. *Acta Cytol.*, **21**, 397.

MEISELS, A., ROY, M., FORTIER, M. & 4 others (1981). Human papillomavirus infection of the cervix. *Acta Cytol.*, **25**, 7.

MIETTINEN, 0. (1976). Estimability and estimation in case-referent studies. *Am. J. Epidemiol.*, **103**, 226.

PUROLA, E. & SAVIA, E. (1977). Cytology of gynecologic condyloma acuminatum. *Acta Cytol.*, **21**, 26.

RAWLS, W.E. & ADAM, E. (1977). Herpes simplex virus and human malignancies. In: *Origins of Human Cancer*. (Eds. Hiatt *et al.*). New York: Cold Spring Harbor Laboratory. p. 1133.

REID, R., LAVERTY, C.R., COPPLESON, M., WIWATWONG, I. & HILLS, E. (1980). Noncondylomatous cervical wart virus infection. *Obst. Gynecol.*, **55**, 476.

REID, R., STANHOPE, C.R., HERSCHMAN, B.R., BOOTH, E., PHIBBS, G.D. & SMITH, J.P. (1982). Genital warts and cervical cancer. *Cancer*, **50**, 377.

SHEVCHUK, M.M. & RICHARD, R.M. (1982). DNA content of condyloma acuminatum. *Cancer*, **49**, 489.

SHOKRI-TABIBZADEH, S., KOSS, L.G., MOLNAR, J. & ROMNEY, S. (1981). Association of human papillomavirus with neoplastic process in the genital tract of four women with impaired immunity. *Gynecol. Oncol.*, **12**, S129.

SPRIGGS, A.I. (1974). Cytogenetics of cancer and precancerous states of the cervic uteri. In: *Chromosomes and Cancer* (Ed. German). New York: Wiley. p. 423.

SPRIGGS, A.I., BUTLER, E.B., EVANS, D.M.D., GRUBB, C., HUSAIN, O.A.N. & WATCHTEL, G.E. (1978). Problems of cell nomenclature in cervical cytology smears. *J. Clin. Pathol.*, **31**, 1226.

SYRJANEN, K.J., HEINONEN, U-M. & KAURANIEMI, T. (1981). Cytologic evidence of the association of condylomatous lesions with dysplastic and neoplastic changes in the uterine cervix. *Acta Cytol.* **25**, 17.

TOOZE, J. Editor *The Molecular Biology of Tumour Viruses*. Part 2: DNA tumour viruses. Cold Spring Harbor Publications, Cold Spring Harbor Laboratory, New York, 1980.

WATERSON, A.P. (1982). Human cancers and human viruses. *Br. Med. J.*, **284**, 446.

ZUR HAUSEN, H. (1982). Human genital cancer: synergism between two virus infections or synergism between a virus infection and initiating events? *Lancet*, **ii**, 1370.


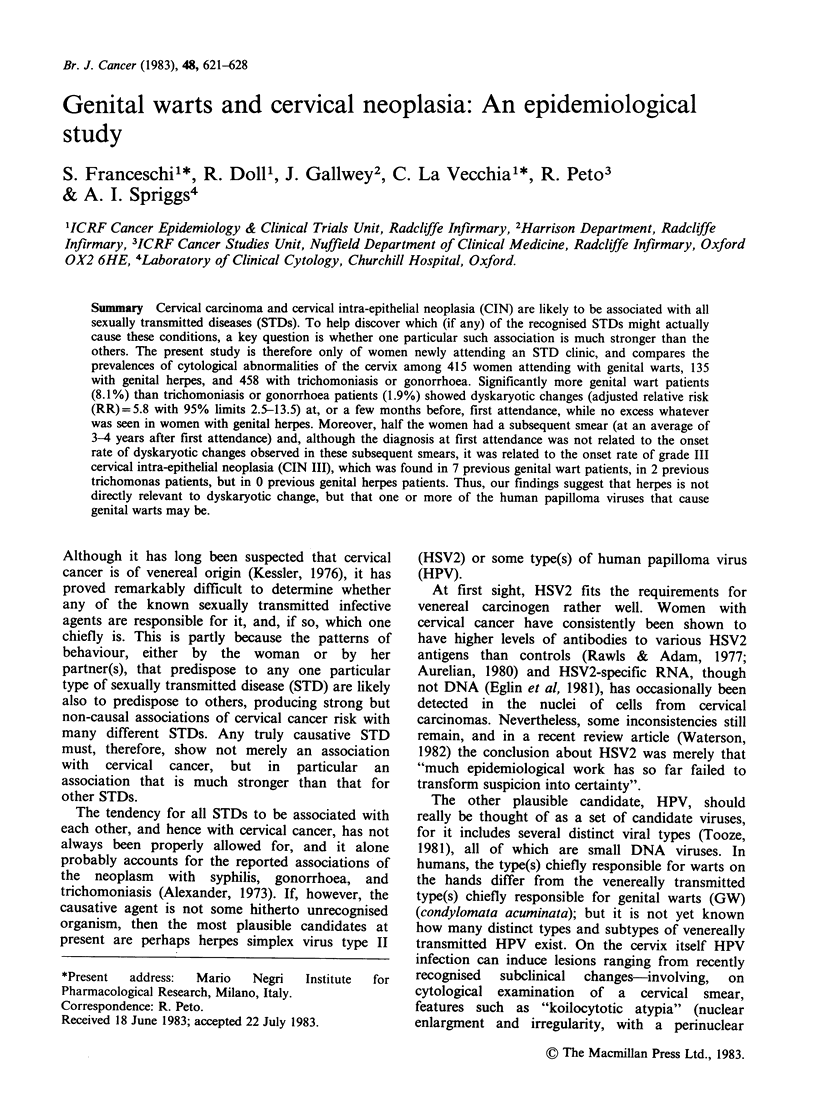

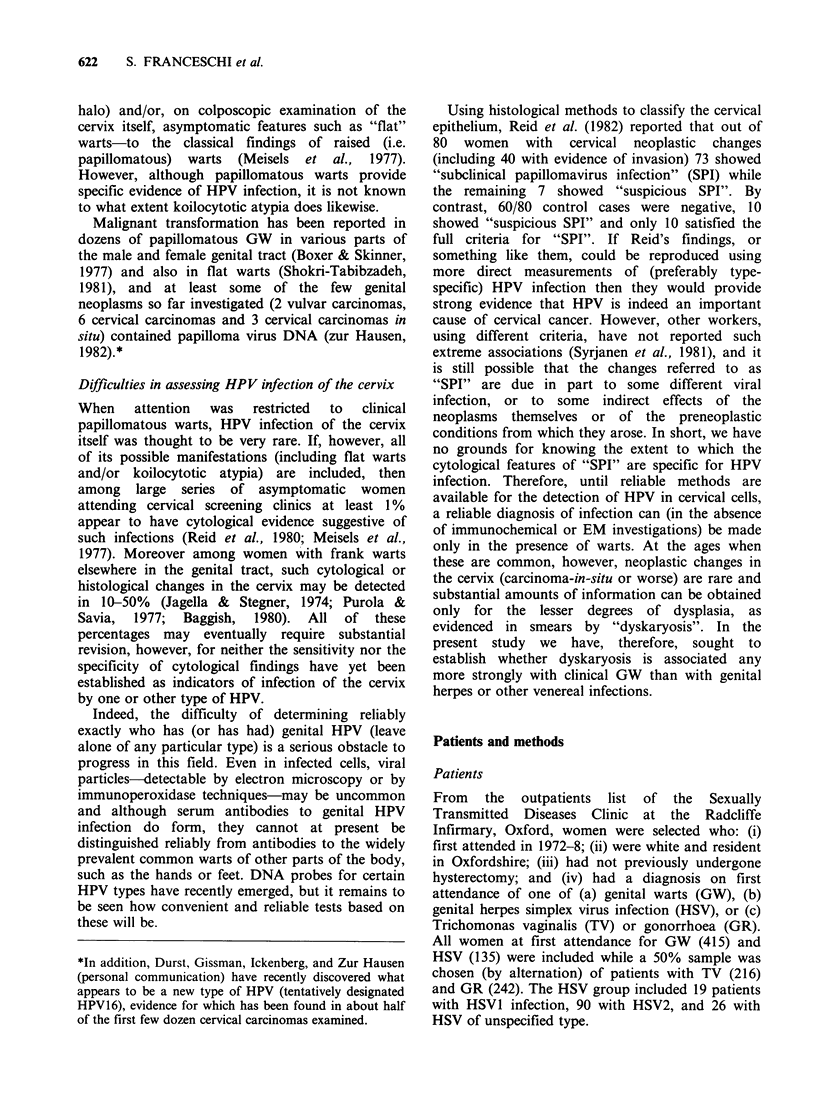

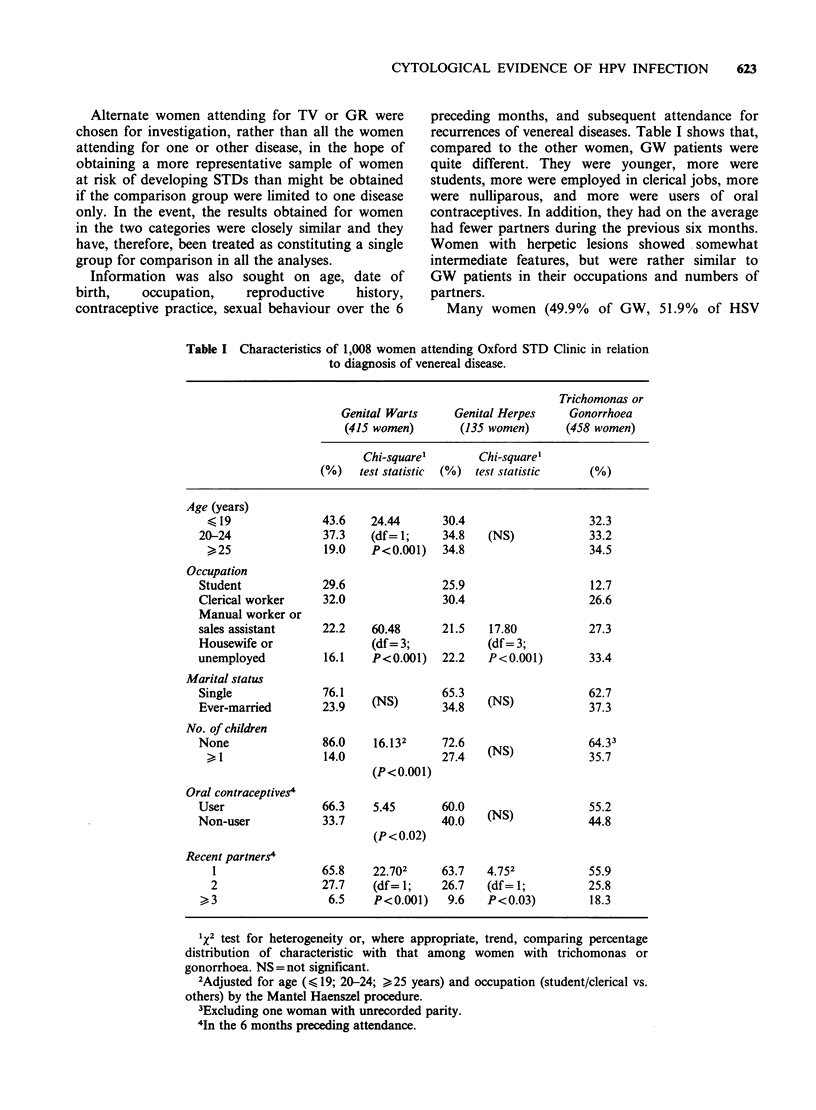

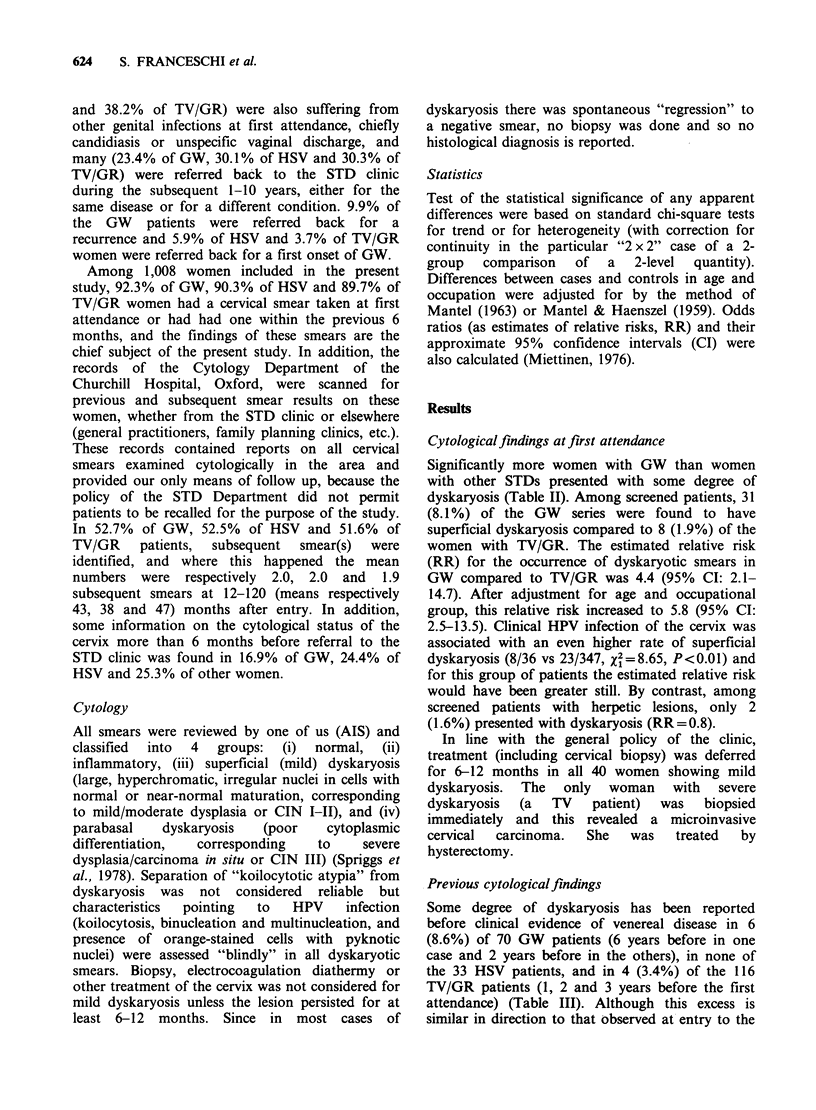

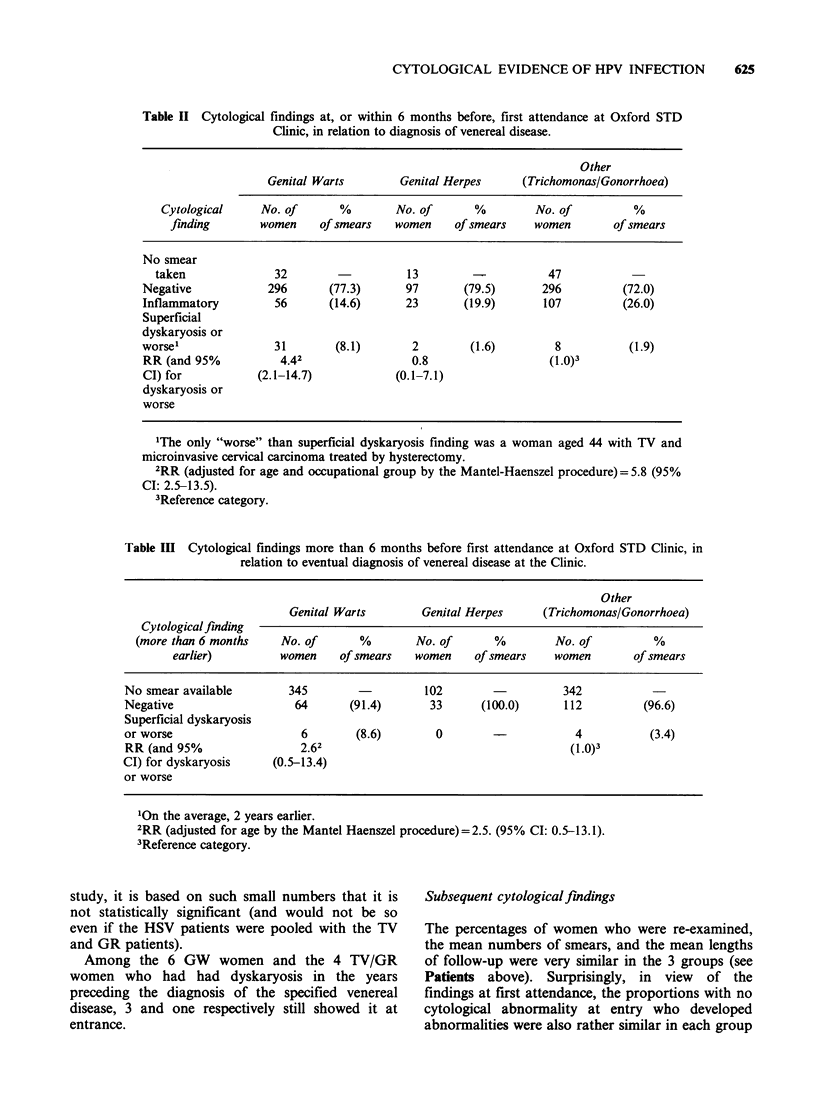

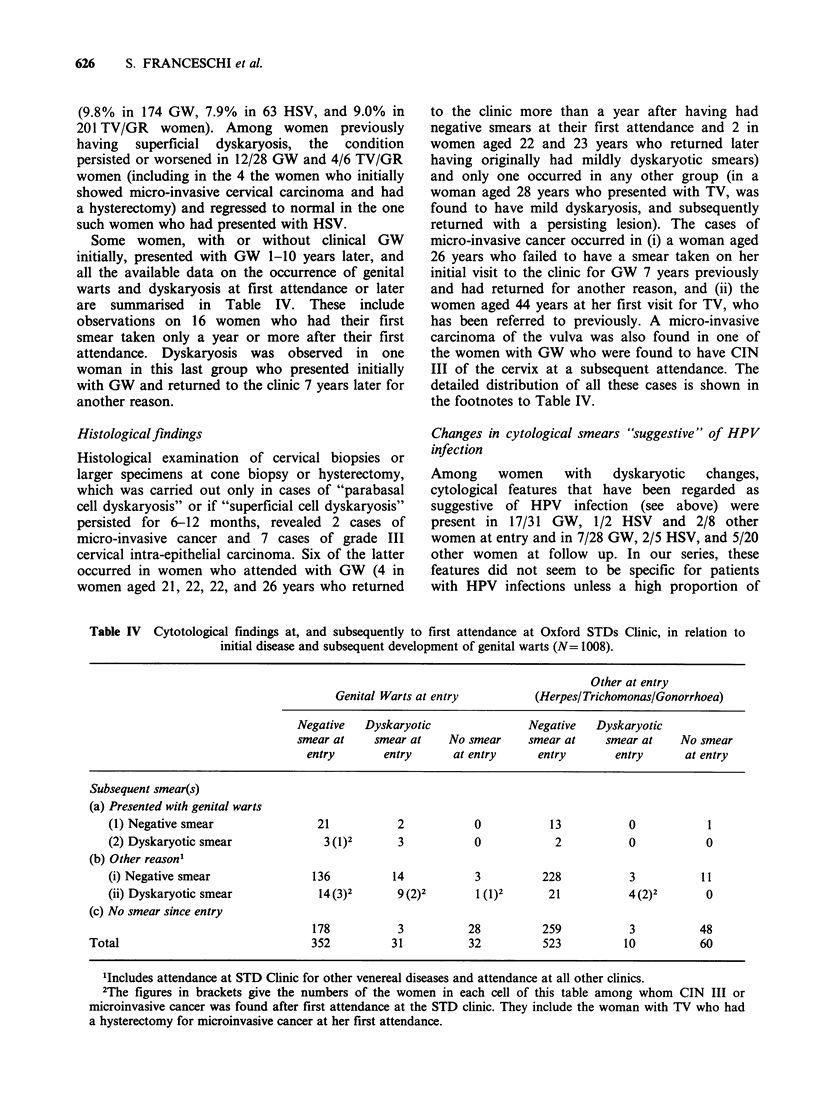

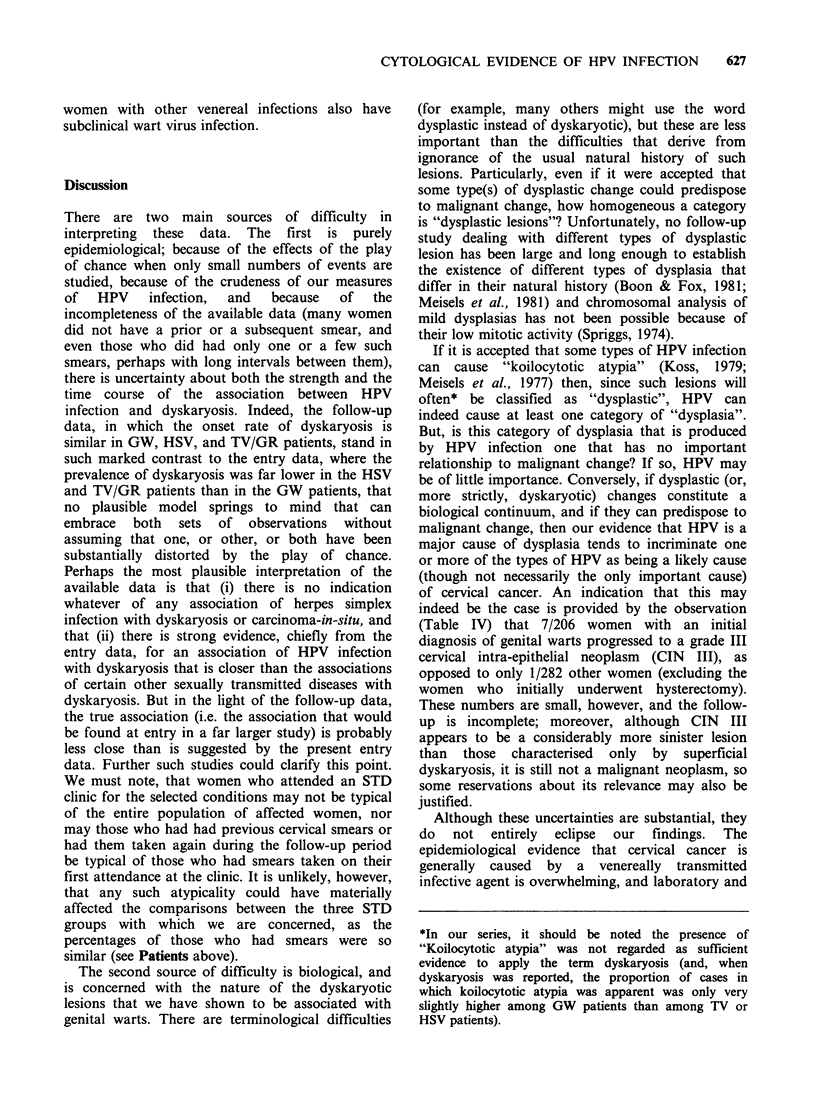

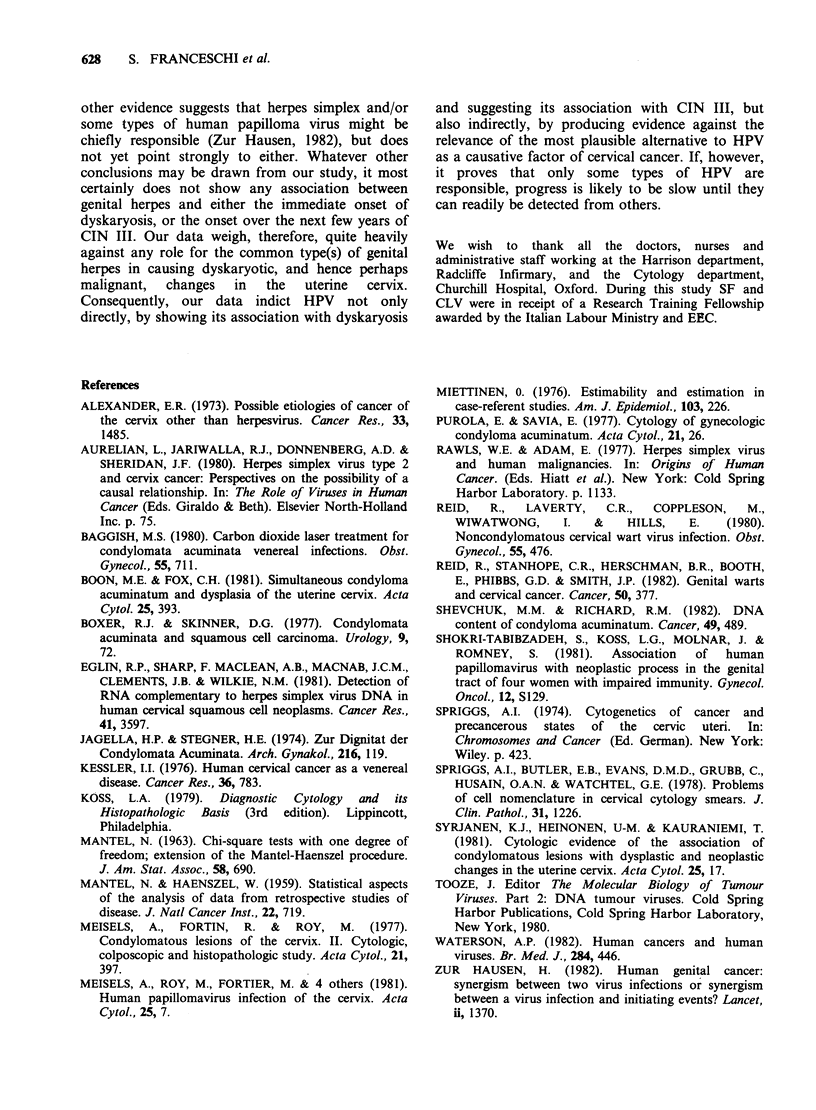


## References

[OCR_00775] Alexander E. R. (1973). Possible etiologies of cancer of the cervix other than herpesvirus.. Cancer Res.

[OCR_00788] Baggish M. S. (1980). Carbon dioxide laser treatment for condylomata acuminata venereal infections.. Obstet Gynecol.

[OCR_00793] Boon M. E., Fox C. H. (1981). Simultaneous condyloma acuminatum and dysplasia of the uterine cervix.. Acta Cytol.

[OCR_00798] Boxer R. J., Skinner D. G. (1977). Condylomata acuminata and squamous cell carcinoma.. Urology.

[OCR_00805] Eglin R. P., Sharp F., MacLean A. B., Macnab J. C., Clements J. B., Wilkie N. M. (1981). Detection of RNA complementary to herpes simplex virus DNA in human cervical squamous cell neoplasms.. Cancer Res.

[OCR_00810] Jagella H. P., Stegner H. E. (1974). Zur Dignität der Condylomata acuminata. Klinische, histopathologische und cytophotometrische Befunde.. Arch Gynakol.

[OCR_00814] Kessler I. I. (1976). Human cervical cancer as a venereal disease.. Cancer Res.

[OCR_00828] MANTEL N., HAENSZEL W. (1959). Statistical aspects of the analysis of data from retrospective studies of disease.. J Natl Cancer Inst.

[OCR_00844] Miettinen O. (1976). Estimability and estimation in case-referent studies.. Am J Epidemiol.

[OCR_00886] (1978). Problems of cell nomenclature in cervical cytology smears. Recommendations of a working party of the British Society for Clinical Cytology.. J Clin Pathol.

[OCR_00848] Purola E., Savia E. (1977). Cytology of gynecologic condyloma acuminatum.. Acta Cytol.

[OCR_00858] Reid R., Laverty C. R., Coppleson M., Isarangkul W., Hills E. (1980). Noncondylomatous cervical wart virus infection.. Obstet Gynecol.

[OCR_00864] Reid R., Stanhope C. R., Herschman B. R., Booth E., Phibbs G. D., Smith J. P. (1982). Genital warts and cervical cancer. I. Evidence of an association between subclinical papillomavirus infection and cervical malignancy.. Cancer.

[OCR_00869] Shevchuk M. M., Richart R. M. (1982). DNA content of condyloma acuminatum.. Cancer.

[OCR_00873] Shokri-Tabibzadeh S., Koss L. G., Molnar J., Romney S. (1981). Association of human papillomavirus with neoplastic processes in the genital tract of four women with impaired immunity.. Gynecol Oncol.

[OCR_00892] Syrjänen K. J., Heinonen U. M., Kauraniemi T. (1981). Cytologic evidence of the association of condylomatous lesions with dysplastic and neoplastic changes in the uterine cervix.. Acta Cytol.

[OCR_00904] Waterson A. P. (1982). Human cancers and human viruses.. Br Med J (Clin Res Ed).

[OCR_00908] zur Hausen H. (1982). Human genital cancer: synergism between two virus infections or synergism between a virus infection and initiating events?. Lancet.

